# Livelihoods, Technological Constraints, and Low-Carbon Agricultural Technology Preferences of Farmers: Analytical Frameworks of Technology Adoption and Farmer Livelihoods

**DOI:** 10.3390/ijerph182413364

**Published:** 2021-12-18

**Authors:** Dandan Zhao, Hong Zhou

**Affiliations:** 1 School of Business, Jingling Institute of Technology, Nanjing 211169, China; 2 School of Business, Nanjing University, Nanjing 210023, China; 3 Grain Research Center, Nanjing Agricultural University, Nanjing 210005, China; zhouhong@njau.edu.cn

**Keywords:** carbon neutrality, farmers’ livelihood, low-carbon technology, preference

## Abstract

In the context of achieving carbon neutrality, it is scientifically important to quantitatively explore the relationships among livelihoods, technological property constraints, and the selection of low-carbon technologies by farmers to promote agricultural modernization and carbon neutrality in the agricultural sector of China. Based on the scientific classifications of farmer capital and low-carbon agricultural technologies, a farmer technology selection theory model considering capital constraints was developed in this study. Microcosmic survey data were collected from farmers in the Jiangsu province for empirical testing and analyses. A total of four low-carbon technologies related to fertilizer usage and three types of farmers’ livelihoods and their relationships were examined by using a logistic model. The results showed the existence of a significant coupling relationship between the intrinsic decision mechanism involved in selecting low-carbon agricultural technology and the properties of low-carbon agricultural technology for different types of farmers. Significant differences exist in the selection of different low-carbon technologies among large-scale farmers, mid-level part-time farmers, and low-level (generally small) part-time farmers. (1) When selecting technology, large-scale farmers are more inclined to accept capital-intensive, low-carbon technologies, such as new varieties, straw recycling, soil testing, and formulated fertilization. Mid-level part-time farmers are more inclined to accept capital intensive, labor saving, or low risk low-carbon agricultural technologies. In contrast, low-level part-time farmers are inclined to accept labor intensive technologies to reduce capital constraints and agricultural risks. (2) Large-scale farmers and low-level part-time farmers are influenced by household and plot characteristics, while mid-level part-time farmers are more influenced by plot characteristics. (3) Households with capital constraints created by differentiated livelihoods face challenges adopting capital-intensive low-carbon agricultural technologies, such as straw recycling, new varieties, soil testing, and formulated fertilization. However, farmers with stronger constraints in the areas of land and labor are more inclined to accept labor-saving technologies, such as soil testing and formulated fertilization technology. Moreover, farmers with stronger risk preferences tend to accept high-risk technologies, such as new technologies like straw recycling. The results of this study can provide a scientific basis for formulating carbon emission reduction policies and low-carbon technology policies for the agricultural sector.

## 1. Introduction

On 22 September 2020, the Chinese government proposed to the 75th Session of the United Nations General Assembly that “China will increase its nationally determined contribution, adopt more effective policies and measures, strive to peak carbon dioxide emission by 2030, and achieve carbon neutrality by 2060” (hereinafter referred to as the “dual carbon” goal). According to the fourth assessment of the United Nations Intergovernmental Panel on Climate Change, agricultural carbon emissions account for 13.5% of global anthropogenic emissions. Agriculture has become the second largest source of greenhouse gas emissions [1]. In China, this proportion is nearly 17%, with an increasing annual trend [2]. This has led to uncertainties with respect to realizing the “dual carbon” goal in China.

Related data indicate that the current productive capacity of cultivated land in China has experienced a continuous decline in soil fertility, with soil acidification occurring in the south and salinization occurring in the north. This is leading to a basic decline in the soil fertility of cultivated land. Fertilizer application and other technologies that have traditionally improved production and efficiency have generally become the primary approach for ensuring stable and increased grain yields in recent years [3]. Cultivated land is the basis for grain production, and the green and low-carbon use of cultivated land is required to improve land quality, advance rural revitalization, and increase food security in China. Key factors that can aid in determining whether China can achieve its “dual carbon” goal include the promotion of agricultural green development (so-called ‘green agriculture’ or ‘sustainable agriculture’, indicating an environment-friendly agriculture characterized by high production efficiency, low carbon emissions, and low contaminant release) and the low-carbon transformation of agricultural production; transforming agricultural development from high-carbon to low-carbon; and reducing agricultural greenhouse gas emissions.

As the key drivers of agricultural production behavior, farmers directly determine the amount of agricultural carbon emissions. Different low-carbon technologies significantly impact the production of agricultural greenhouse gases, with differences in the risk to the carbon footprint [4]. Farmers with different livelihood characteristics and capital resources make different adoption decisions about selecting low-carbon technologies [5]. When farmers select low-carbon agricultural technologies, they generally consider their own capital constraints, technological risks, and other constraints. This can lead to a technology selection bias. This highlights the need to understand the technological path, carbon reduction measures, and policy implementation dynamics with respect to future agriculture development in China. This involves understanding the selection paths and preferences with respect to low-carbon technologies based on different farmer livelihood levels, and the constraints, the factors, and the impacts they face.

Previous studies on farmer adoption of low-carbon agricultural technologies are mainly focused in the following areas. The first area relates to the farmers’ willingness to adopt these technologies and the factors impacting these behaviors, which include attitudes [6], livelihood decisions [7], subjective standards [8,9], perceived behavioral control [10], and contract enterprise participation. When these factors are higher, farmers tend to be more willing to adopt low-carbon technologies. Household-related factors impacting farmers’ low-carbon technology adoption behaviors include their education level [11], their environmental understanding and perceptions [12], the age of the head of household [13], and their gender [14,15]. Land-based factors impacting low-carbon technology adoption by farmers include land scale [16,17], land ownership [18], natural capital [14], and policy factors [19]. Risk perceptions have also been found to play an important role, including the influence of risk tolerance [20], the degree of risk aversion [21], and the perceived risk [17,22,23].

The second key research area relates to the low-carbon agricultural technology selection bias. Scholars have analyzed farmer selections of low-carbon production technologies, including no-tillage [24], straw recycling [25,26], organic fertilizer and nitrogen fertilizer [27,28], and other technologies. For example, a researcher used agricultural film and straw treatment as examples to analyze environmental equity, social trust, and low-carbon production behaviors [29]. According to the literature, the carbon emissions of no-tillage methods were measured as a tool for reducing nitrogen fertilizer application, as well as technologies for planting and breeding in paddy fields [3]. Moreover, the reduction in pesticide application to analyze low-carbon adoption behaviors of farmers was also studied previously [25].

Previous studies have researched the utilization and adoption of agricultural low-carbon technologies from multiple perspectives; however, they have mainly analyzed the adoption of one or two low-carbon technologies and have not discussed broader choices of low-carbon technologies. Further, the impact of different livelihoods on the selection of low-carbon methods for use on cultivated land have not been investigated to date. Finally, previous analyses of the factors impacting technology selection have been relatively general, and most of them do not include the support of a theoretical model. This makes it difficult for the results to guide practice, in particular when the basic assumptions and applicable conditions of the model are ambiguous. Based on this, the perspectives of different livelihoods of farmers, as well as combining individual characteristics, family properties, the land characteristics of farmers, the factors impacting farmer livelihoods, and the factors impacting the selection of low-carbon technologies, supported by a classification framework of typical low-carbon technologies as well as empirical tests, were systematically analyzed in this study.

The main objectives of this study are as follows: (1) to classify low-carbon agricultural technology types by combining the carbon footprint literature and integrating capital, labor factor input intensity, and technological risk; (2) to develop a theoretical model of low-carbon agricultural technology selection considering the properties and capital constraints of farmers and technological risk assessments, and to clarify the mutual feedback coupling the relationship between these variables and the applicable conditions; and (3) to reveal the preference of Chinese farmers with different livelihood types and levels of properties when selecting different agricultural low-carbon technologies. The intent of the study is to contribute to a deeper understanding of the behavior logic underlying the selection of low-carbon technology by Chinese farmers, and the institutional challenges they face.

## 2. Theoretical Analysis Framework

In an environment of incomplete and asymmetric market information, farmers and technology types are divided based on the factors that affect farmers’ technology adoption, such as land, properties, capital, labor resources, and risk preference. Specifically, according to the livelihood differentiation, farmers are divided into three types according to land, capital, labor resources, and risk preference: large-scale farmers, mid-level part-time farmers, and low-level part-time farmers. Large-scale farmers refer to large plantation farmers that transfer land into large-scale management. In this study, farmers in the top 10% of cultivated land area with household management in their counties are defined as large-scale farmers. Mid-level part-time farmers are farmers whose area of cultivated land management does not reach the level of large-scale farmers, and who have a household non-agricultural income proportion exceeding 70%. In contrast, low-level part-time farmers are farmers whose cultivated management scale does not reach the level of large-scale farmers and who have a household non-agricultural income proportion of less than 30%. In the study, the rural market is not fully developed in China, significant differences exist in production factor constraints and risk preferences of these three types of farmers, as presented in Table 1.

(1) Large-scale farmers: these farmers transfer more land, have a larger scale of production, and a stronger risk resistance. However, they face greater cost pressure and pressure from losses, with lower risk aversion. In terms of labor, these farmers can hire workers during the busy farming season; however, agricultural production is affected by seasonal labor shortages and rising labor costs in the busy farming season, strongly restricting worker employment. In terms of capital, although large-scale farmers have some financial strength, they have higher credit constraints due to their large agricultural production scale and lack of mortgage and guarantee conditions.
(2) Mid-level part-time farmers: these farmers face significant labor constraints, but due to a higher non-agricultural income, they have lower capital constraints for agricultural production, and therefore are more resistant to risk and are less risk averse.
(3) Low-level part-time farmers: these farmers are more risk averse and are strongly restricted by capital; however, these farmers have more available and relatively abundant labor compared to land resources.

The factor input intensity and the risk associated with low-carbon agricultural technologies are closely related to the livelihoods and risk preferences of farmers. As such, this article combined the two properties of factor input intensity and risk, and divided the main agricultural low-carbon technologies into the following four categories: capital intensive–labor stabilizing–high-risk technologies, such as new varieties; capital intensive–labor saving–high-risk technologies, such as straw recycling; capital intensive–labor saving–low risk technologies, such as soil testing and formulated fertilization; and capital stabilizing–labor increasing–low-risk technologies, such as farmyard manure application.

Compared with the old varieties such as straw burning and fertilizer application, adopting the technologies illustrated in the four above-mentioned examples exhibit different characteristics in terms of benefits, uncertainties, labor input, and the capital investment of technology adoption. They are described further as follows:

(1) New varieties (capital intensive–labor stabilizing–high-risk technology): the role of new technologies is mainly to reduce carbon emissions; however, they may increase the uncertainty of outputs (crop yields and quality). The demand for labor remains unchanged, but it may increase capital investments in agricultural production.
(2) Straw recycling (capital intensive–labor saving–high-risk technology): straw recycling technology can eliminate the greenhouse gas emissions caused by straw burning and can indirectly reduce the input of chemical fertilizer. However, this requires a long time for the response. Straw recycling technology mostly uses machines to grind straw and turn it into soil, which can save agricultural labor and increase capital investments compared with burning behaviors without recycling. However, it is uncertain whether straw recycling can increase crop yields, because an excessive or uneven amount of straw recycling can cause the growth of soil microorganisms and crop seedlings have to compete for nutrients. Further, eggs and bacteria carried by straw cannot be killed in the grinding process, which may leave latent dangers from pests and disease.
(3) Soil testing and formulated fertilization (capital intensive–labor saving–low risk technology): soil testing and formulated fertilization technology requires a higher capital investment compared with ordinary fertilizer. However, it is a more low-carbon and ecological approach, and reduces uncertainties associated with outputs. Soil testing and formulated fertilization are known to increase long-term yields.
(4) Farmyard manure (capital stabilizing–labor increasing–low risk technology): compared with ordinary fertilizers, farmyard manure technology is greener and is a more low-carbon emission technology. Its significant characteristics include increased labor input and reduced risk uncertainty (lower risk). This technology generally does not increase the costs of agricultural production.

Considering the constraints faced by farmers and the technical properties related to farmer activities, it has been hypothesized that the three types of farmers introduced above have different preferences for the four low-carbon technologies (Table 2).

Based on the above-mentioned theoretical analysis, this study proposed the following research hypotheses:

**Hypothesis** **1.** *Large-scale farmers perform specialized production activities, and agricultural income is their main source of income. They have lower levels of risk aversion and operate at a larger scale and are strongly constrained by capital and labor. When selecting low-carbon agricultural technologies, they tend to accept new varieties, straw recycling, soil testing, and formulated fertilization technologies*.

**Hypothesis** **2.** *With non-agricultural income as their main source of income, mid-level part-time farmers have weaker capital constraints and lower levels of risk aversion but face strong agricultural labor constraints. They prefer labor saving or non-labor increasing agricultural low-carbon technologies, including new varieties, straw recycling, soil testing, and formulated fertilization technologies*.

**Hypothesis** **3.** *Low-level part-time farmers have weaker labor constraints, stronger capital constraints, and higher levels of risk aversion. They can improve agricultural low-carbon utilization by applying farmyard manure that requires labor input but have little motivation to adopt other capital-intensive technologies*.

## 3. Data Source, Variable Selection, and Model Setting

### 3.1. Data Source

Data used in this study were collected using a household survey of grain growers in the Jiangsu province in July 2021. To ensure sample diversity and representativeness, the survey area was mainly concentrated in the following four counties (cities): Rugao, Gaoyou, Xinghua, and Sheyang. Compared to other regions, this region has a higher agricultural production level, faster urbanization, better economic development, and more common participation by farmers in non-agricultural employment. When focusing on the livelihood differences between farmers, the low-carbon agricultural choices of grain growers are representative.

Stratified random sampling and stratified sampling methods were adopted for this survey. Data presented in this study were collected by using the results of a farmer’s survey in the Jiangsu province in July 2021. This survey was conducted mainly in four counties (cities), including Rugao, Gaoyou, Xinghua, and Sheyang. In order to reduce errors and uncertainty in the farmer’s survey, each questionnaire was completed by a trained investigator face-to-face. After eliminating some unqualified questionnaires, a total of 307 questionnaires covering 1191 plots and 307 farmers were obtained and used for further analysis. Compared to other regions, investigated regions were, in general, featured by higher agricultural productivity and yield, rapid urbanization, advanced economic development, and a large amount of non-agricultural employment, which benefited the exploration of the relationships between different farmers and low-carbon agricultural technologies. Among the four selected counties (cities), the resident populations of Rugao, Gaoyou, Xinghua, and Sheyang are 1,238,400, 745,900, 1,128,200, and 759,400 in 2020, respectively, and the number of towns are 26, 27, 35, and 21 for the four counties, respectively. Overall, 3–4 towns of each county (city), and 2–3 administrative villages of each town, were selected and related research was conducted by using this survey. Furthermore, 10–15 farmers of each administrative village were randomly investigated. The database was established by using Excel 2010 software. The statistical analysis was carried out by using stata15.0 software. Basic information of surveyed farmers has been shown below (Table 3).

### 3.2. Model Setting

In this study, farmer survey data were used to test the hypotheses proposed in the theoretical model about the relationships between farmer livelihood and low-carbon agricultural technology selection. Three models were conducted to test the hypotheses introduced above using the logistic method. First, the relationships between different farmers (large-scale farmers, mid-level part-time farmers, and low-level part-time farmers) and four low-carbon agricultural technologies were discussed (Section 4.1 and Section 4.2). Second, the main driving forces of the specific farmer (large-scale farmers, mid-level part-time farmers, and low-level part-time farmers) influencing the selection of the four low-carbon agricultural technologies were determined (Section 4.3). Finally, the impact of the farmer’s properties on the selection of the four low-carbon agricultural technologies (Section 4.4) was explored. The dependent variable was a binomial classification variable. For instance, in terms of new varieties, when farmers select this low-carbon agricultural technology, then the dependent variable was set as 1, and otherwise it was 0.

First, the theoretical logistic model is represented as follows:

For binary selection behavior, the net benefit (benefit minus cost) of the behavior can usually be summarized by using a “latent variable”. If the net income is greater than 0, select “yes”; otherwise, choose “not to”. The assumed net income is:

(1) y*=xβ+ε 

where the net income y* is the latent variable and is unpredictable. The above-mentioned formula is also called the “index function”. The equation is represented as follows:

(2) $$y=\left\{ \begin{array}{l} 1,\;if\;y*>0\\ 0,\;if\;y*\leq 0 \end{array}\right.\;$$

Second, the measurement model of differences in farmer livelihood and the selection of agricultural low-carbon technologies is constructed as follows:

(3) $$y_{i}=\beta_{0}+\sum_{i=1}^{n}\beta_{ij}X_{ij}+\sum_{i=1}^{n}\gamma_{ij}Z_{ij}+\varepsilon_{i}\;$$

In this Equation (3), *y_i_* is the dependent variable, *i* = 1, 2, 3, and 4, indicating the four low-carbon agricultural technologies, namely new varieties, straw recycling, soil testing, and formulated fertilization. If this technology is selected, *y_i_* is 1, otherwise it is 0. With regard to first model, the main independent variable, *X*, included large-scale farmers and mid-level part-time farmers. *Z* denotes the control variable, including a group of control variables affecting the selection of agricultural low-carbon technologies by farmers (besides farmer type). These control variables mainly included the factors of distance from the village to the town, the intensity of agricultural machinery popularization in the village, the degree of circulation of village land, and whether agricultural machinery service stations were present or not. For the second model, the main variable, *X*, included age, education level, health level, whether they were the village cadre or not, and whether the farmer is a land transfer. *Z* denotes the control variable, including the lease term, land rent, and the number of plots. For the third model, the main variable, *X*, included capital, risk preference, and land-labor resources. The control variables are the same as those in the second model. An unobservable disturbance term, *εi*, was also controlled in this model.

### 3.3. Selection of Variables

Dependent variable: the major dependent variable was the farmers’ selection behaviors with respect to agricultural low-carbon technologies. Low-carbon technologies were divided into the following four categories: capital intensive–labor stabilizing–high-risk new varieties, capital intensive–labor saving–high-risk straw recycling, capital intensive–labor saving–low-risk soil testing and formulated fertilization, and capital stabilizing–labor increasing–high-risk farmyard manure.

Independent variable: the major independent variable was farmer livelihood. Farmer livelihood levels are defined based on the study by Zhang and Qian [30] and included large-scale farmers, mid-level part-time farmers, and low-level part-time farmers. Large-scale farmers referred to large plantation farmers who transferred land into large-scale management. In this study, the large-scale farmers in the top 10% of agricultural areas with household management in their counties are defined as large-scale farmers. Moreover, other variables such as the head of household information, family characteristics, plot information, and other independent variables were also included. Table 4 lists the definitions and descriptive statistics of variables used in the model.

## 4. Results

### 4.1. Farmer Livelihood and the Selection of Agricultural Low-Carbon Technologies

Table 5 lists the farmer livelihoods and the selection of agricultural low-carbon technologies. Different farmers made significantly different technology selections. For example, large-scale farmers’ adoption of the capital intensive–labor stabilizing–risk-increasing technology (new varieties) was significantly higher compared to that of other farmers, and it passed the significance test at the 10% level; however, they have a lower willingness and proportion of farmyard manure application. The proportion of mid-level part-time farmers selecting the four low-carbon technologies was 52.43, 71.35, 8.1, and 15.13 for new varieties, straw recycling, soil testing/formulated fertilizer, and manure, respectively. Among these, the proportion selecting straw recycling, soil testing, and formulated fertilization was significantly lower compared to farmers with other livelihoods, while the proportion selecting farmyard manure application was moderate. However, the proportion of low-level part-time farmers selecting new technologies was the lowest (42.86%), and the proportion selecting farmyard manure application was the highest (21.43%).

### 4.2. The Regression Results of Farmer Livelihood and the Selection of Low-Carbon Agricultural Technologies with Different Properties as Well as Impact Factors

Table 6 lists the estimated results and average marginal effects derived from the logistic model. The Equation (3) was applied to analyze the factors impacting the selection of four different low-carbon technologies. Results showed that after controlling for regional (county) heterogeneity, farmer livelihoods showed significant differences when selecting different low-carbon agricultural technologies. With respect to key variables, large-scale farmers and mid-level part-time farmers were more likely to select capital intensive–labor saving–high-risk technology (straw recycling) in contrast to low-level part-time farmers; the difference was significant at the 1% significance level. Large-scale farmers selected capital intensive–labor stabilizing–high-risk technology (the new variety) and capital intensive–labor saving–low-risk technology (soil testing and formulated fertilization) at significantly higher levels than the low-level part-time farmers; this result was significant at a 10% significance level. There was no significant difference from the mid-level part-time farmers. Although the low-level part-time farmers selected the first three technologies at significantly lower levels than the other two types of farmers, they showed a higher preference for the capital stabilizing–labor increasing–low-risk technology (farmyard manure application); this result was statistically significant at the 1% level.

### 4.3. The Analysis of Factors Impacting the Selection of Low-Carbon Agricultural Technology by Different Types of Farmers

#### 4.3.1. Large-Scale Farmers and the Selection of Agricultural Low-Carbon Technologies

The above-mentioned results showed that different farmers made significantly different selections with respect to different low-carbon agricultural technologies. Based on the sub sample data of different farmers, the relationship between farmers and the choice of four low-carbon technologies and its influencing factors is discussed in this study.

This further analyzes the factors that most affect these technology selections for different types of farmers. Table 7 presents the results of the regression analysis of the factors impacting large-scale farmers and their selection of low-carbon agricultural technologies. First, the age of large-scale farmers and the position of village cadres exhibited a significant positive impact on the adoption of new varieties. However, land rent and the quantity of land showed negative effects. Second, the education level of large-scale farmers exhibited a significant positive effect on the adoption of straw recycling technology, with a mean marginal effect coefficient of 0.005. This indicates that large-scale farmers with a higher education level were more likely to adopt straw recycling. Similarly, the number of plots showed a significant negative impact on the adoption of this technology. Third, the health status of large-scale households, whether they are village cadres, whether they are land transfer farmers, and the lease term of land showed positive effects on the application of soil testing and formulated fertilization technology. Fourth, the basic characteristics and properties of large-scale farmers had negative effects on farmyard manure application, with large-scale farmers being generally averse to this technology because of its high labor requirements, terrible smell, low nutrients, dispersed sources, and uncertainties [26].

When considering the four low-carbon technologies (except the application of farmyard manure), the individual characteristics and properties of large-scale farmers, in particular, their age and education level, showed significant positive effects on the adoption of low-carbon technologies. Most of the plot characteristics had a negative impact on the adoption of the four low-carbon technologies, in particular, the number of plots and land rent. The number of plots somewhat inhibited large-scale farmers from adopting new varieties and straw recycling technologies. This is attributed to the fact that the number of plots is too large for contiguous management, thus leading to the increase in the input costs of agricultural production. The selection of low-carbon agricultural technology by large-scale farmers is affected by household information and plot characteristics.

#### 4.3.2. Mid-Level Part-Time Farmers and the Selection of Agricultural Low-Carbon Technologies

Table 8 summarizes the regression relationship between mid-level part-time farmers and their selection of low-carbon agricultural technologies. The regression results for the head of the household information showed that health and their education level exhibited relatively low negative effects on the selection of some low-carbon technologies; however, other information regarding the head of the household played a positive role in promoting the application of the four low-carbon technologies. In particular, whether a farmer was a land transfer farmer or not, and health, were found to be two factors that had a significant influence. For mid-level part-time farmers, the lease term had a significant positive effect on the adoption of the four low-carbon technologies. This indicates that the longer the lease term of the mid-level part-time farmer, the higher the tendency to adopt low-carbon agricultural technologies. For the three technologies, including the new varieties, straw recycling, soil testing, and formulated fertilization, the land rent and the number of plots showed negative impacts for mid-level part-time farmers. This trend was similar to large-scale farmers. This also indicates that the adoption of major low-carbon technologies was mainly affected by the household income level. However, for the application of farmyard manure, the land characteristics of mid-level part-time farmers showed a significant positive effect on the traditional tillage. In contrast to large-scale farmers, mid-level part-time farmers reported mainly using their cultivated land to meet basic food needs due to their smaller number of plots and flexible lease term. However, they scientifically understood the improvements in grain quality possible with organic fertilization and were willing to take actions to adopt the technology.

#### 4.3.3. Low-Level Part-Time Farmers and the Selection of Agricultural Low-Carbon Technologies

Table 9 presents the analysis of the factors impacting the four low-carbon agricultural technology selections by low-level part-time farmers. The regression coefficients with respect to these farmers’ selection of new variety technologies was not significant, indicating that low-level part-time farmers were not very interested in selecting new varieties and exhibited a lower selection preference. For straw recycling, whether the farmer was a land transfer farmer, and the lease term, had significant positive effects on whether low-level part-time farmers selected the technology; status as a land transfer farmer had a greater impact, indicating that the low-level part-time farmers with land transfer properties showed a stronger preference for straw recycling technology.

In terms of soil testing and formulated fertilization, the negative average marginal effect coefficient denoted a negative impact of land rent on the selection of these technologies (the coefficient was −0.04). This indicates that when other factors were constant, the preference of low-level part-time farmers to select soil testing and formulated fertilization technologies increased by 0.04 units when the land rent decreased by 1 unit. However, only the number of plots in the properties of low-level part-time farmers positively impacted the selection of farmyard manure. This also indicated that the low-level part-time farmers consisted of a larger number of plots, somewhat increasing the costs of agricultural production and low-carbon technology. Therefore, the number of plots was the main factor affecting the application of farmyard manure for low-level part-time farmers.

### 4.4. The Regression Results of Family Properties and Low-Carbon Agricultural Technology Property Characteristics

The above-mentioned research indicates that, for different types of farmers, the head of the household and plot characteristics have significant reverse effects on the selection of the four low-carbon technologies studied. However, whether or not family properties impact the selection of low-carbon agricultural technologies based on constraints facing different farmer households and the influence of family properties characteristics on the selection of low-carbon agricultural technologies by farmers at different farmer livelihood levels was further observed. This included the examination of the influence of capital, risk preference, and land-labor resources on the selection of technologies that are either capital-intensive or capital-stabilizing, labor-increasing or labor-saving, or high-risk or low-risk. Table 10 presents the goodness of fit test of the model that is significant at 1% and 5% levels, indicating that the model effectively reflected the explained variables. The following main results were found.

First, the degree of influence between the capital of the farmer household and the capital-biased low-carbon technology was observed. Table 10 summarizes that capital significantly increased the application of straw recycling and new variety technologies at a 1% statistical significance level; and straw recycling and new variety technologies and low-carbon agricultural technologies required more capital investment than traditional technologies. The regression results associated with farmyard manure application technology show that capital was significantly negative at a 1% significance level, which also indicates that farmyard manure application technology generally does not require too much capital. Instead, it mainly depends on the input of agricultural labor to achieve the effect. The evolution of capital created by farmer livelihood differentiation shows a strong constraint on low-carbon agricultural technologies with capital-intensive properties. Farmers with more abundant capital tend to have higher labor opportunity costs and prefer technologies that can achieve low-carbon agricultural utilization by increasing their capital outlay.

Second, a degree of influence between household land and labor resources and labor-biased low-carbon technologies was observed. The regression results presented in Table 10 indicate the existence of significant heterogeneity in family properties with respect to the application of labor-increasing farmyard manure, labor-saving soil testing, and formulated fertilization. The family’s land-labor resources significantly increased the selection of labor-increasing technology (farmyard manure application) at the 5% significance level. and significantly inhibited the selection of labor-saving technology (soil testing and formulated fertilization) at the 5% statistical significance level. This also indicated that farmers with stronger land-labor resource constraints were more likely to accept labor-saving technologies; otherwise, they possibly selected labor-increasing technologies to realize agricultural low-carbon utilization.

Finally, an influencing relationship was observed between risk preference and risk-biased low-carbon technologies. Farmers in agricultural production mainly consider the maximization of household income. Different low-carbon technologies may stabilize yields, increase low-carbon utilization, and protect cultivated land. However, from a risk perspective, the use of new varieties has uncertain impacts on yields; straw recycling only increases yields if used over the long term, and soil testing and formulated fertilization reduces the uncertainty of yields. Therefore, households with different risk preferences reported selecting low-carbon technologies with different risk properties. Table 10 presents that farmer risk preferences led to a significant increase in straw recycling and new variety technologies at a 1% statistical significance level; that is, farmers with higher risk tolerance tended to accept risk-increasing technologies, and vice versa.

## 5. Discussion

### The Differences and Main Factors Impacting the Selection of Low-Carbon Agricultural Technologies by Heterogeneous Farmers

Agricultural labor availability is declining in China; the importance of agriculture in rural families in China is continuously declining. Farmer livelihoods, such as the use or the offer of part-time labor has changed, and encouraging low-carbon agricultural technology has become increasingly difficult in the face of small-farmer management. Small farmers may offer disadvantages with respect to science and technology knowledge, cognition, or agricultural production development [31]. This significantly restricts agricultural carbon emission reduction and the advancement of low-carbon technologies and increases the contribution rate of low-carbon technologies. Overall farmer enthusiasm for adopting new technologies is not high in China. In this study, the questionnaire asking about the selection of four low-carbon agricultural technologies showed that the current low-carbon agricultural technology promotion policy has some level of response, but the response effect needs improvement.

In this study, farmers were divided into different types for classification purposes. Different farmers reported significant differences in their selection of low-carbon agricultural technologies. In a previous study, rural data in the Hubei Province were used and it was concluded that 73% of farmers applied a standard or reduced amount of chemical fertilizer in agricultural production and showed a strong intention to adopt low-carbon approaches [32]. However, differences in technological selection among different farmers were not observed, and scholars analyzed low-carbon production behaviors of Xinjiang cotton farmers but did not consider their differentiation or the part-time status of farmers [33]. The study concluded that farmers were willing and enthusiastic about adopting low-carbon approaches, but it was difficult to understand the behavior logic of different farmers when selecting different technologies. This made it difficult to understand institutional obstacles when advancing agricultural technology, because significantly large differences were observed in the property characteristics of different farmers. As a result, classifying these farmers by livelihood is of great value when advancing agricultural technology.

The results of this study showed that large-scale farmers were more inclined to accept capital intensive–labor stabilizing–high-risk technologies (i.e., new technology), capital intensive–labor saving–high-risk technologies (i.e., straw recycling) or capital intensive–labor saving–low risk technologies (i.e., soil testing and formulated fertilization). The high-risk current farmers were more inclined to choose capital intensive–labor saving–high-risk (straw recycling) or capital intensive–labor saving–low risk (soil testing and formulated fertilization) technologies. Compared with the other two types of farmers, low-level part-time farmers were more inclined to accept the farmyard manure application technology. The conclusion is somewhat consistent with Liu [34]. Applying the perspective of bounded rationality, Liu [34] compared and analyzed the selection of low-carbon technologies by traditional farmers and new management entities. This study found that the new agricultural management entities had a stronger preference for low-carbon new technologies, which also relates to the cultivated land scale of new management entities and the family properties of farmers.

The results of this study differ from those by Zhao et al. [29]. Shaanxi and Gansu found that farmers showed a strong adoption of straw recycling low-carbon technology; however, the study did not differentiate farmer livelihoods. Instead, low-carbon technology behaviors were analyzed from the perspective of the environmental equity perception, considering farmers as one uniform population. The study further concluded that environmental equity perceptions resulted in a significant positive increase in the adoption of straw recycling technology by farmers. However, with the increased differentiation of farmers and the evolution of farmer livelihoods, the property characteristics of heterogeneous farmers have changed and thus the analysis of farmers as a whole is not of strong significance for policy guidance. Overall, the classification and comparison of farmers in this study supports the advancement of low-carbon agricultural technologies for different farmers.

The results of this study further identified that the factors impacting the selection of low-carbon technology by different farmers were mainly influenced by individual characteristics and plot information. First, the health status and whether there was a village cadre for large-scale farmers had positive promotion effects on the selection of new technologies. The health status, whether there was a village cadre, and whether the farmer was a land transfer farmer advanced the adoption of soil testing and formulated fertilization technologies, while the education level advanced the adoption of straw recycling technology. The main reason is that large-scale farmers are households with predominantly agricultural income and are generally more concerned about whether the head of the household and external characteristics add an additional burden, as this may impact the acceptance of low-carbon agricultural technologies.

Second, in addition to the lower negative effects of health status and educational level, other information regarding the head of the household with mid-level part-time farmers played a positive role in advancing the application of four low-carbon technologies. For three technologies (new varieties, straw recycling, and soil testing and formulated fertilization), the land rent and number of plots showed a negative effect on mid-level part-time farmers in low-carbon agricultural selection. Non-agricultural income was dominant for mid-level part-time farmers; as such, methods with the highest efficiency and the lowest labor input were generally adopted when performing low-carbon agriculture technologies.

Finally, with respect to the individual characteristics of low-level part-time farmers, the status of the farmer as a land transfer farmer led to an increase in the acceptance of straw recycling technology; however, other head of the household information had no significant impact on agricultural low-carbon technologies. This study found that with respect to plot characteristics, the lease term increased the acceptance of straw recycling technology; the land rent increased the acceptance of soil testing and formulated fertilization technology, and the number of plots negatively impacted farmyard fertilizer application technology. This also indicates that low-level part-time farmers showed more plots, somewhat increasing the costs of agricultural production and low-carbon technology utilization. Therefore, low-level part-time farmers selected the labor-increasing farmyard manure application technology.

From the perspective of the factors impacting the selection of low-carbon agricultural technologies, Zhang et al. [35] found that individual characteristics were the main factor influencing the selection of low-carbon technologies for large-scale farmers. This study found that among individual characteristics, the education level of large-scale farmers led to the increase in the adoption of low-carbon technologies by farmers. This further confirmed that non-agricultural employment could affect behaviors that impact agricultural carbon emissions shaped by farmers; moreover, part-time farming could promote the low-carbon behaviors of farmers [18,19]. At the same time, previous studies have not classified low-carbon agricultural technologies; instead, they set “whether to adopt low-carbon technologies” as the dependent variable for regression. Many studies have evaluated different low-carbon technologies than those selected in this study. These literature studies evaluated completely different low-carbon technologies, such as the agricultural film and straw recycling technologies adopted by Zhao et al. [29].

In summary, previous studies analyzed the factors impacting the agricultural low-carbon behaviors of farmers from different perspectives, but they did not classify farmers based on their livelihood or the properties of low-carbon technologies. These differences can increase the difficulties involved in studying the selection of low-carbon technologies by farmers and can make it difficult to more specifically present the selections, and the drivers for those selections, of low-carbon technologies by different groups of farmers.

Based on this, when further observing whether there exists a causal relationship between the characteristics of family properties and low-carbon technologies with different properties, an asymmetric deviation is observed between the family property characteristics of farmers and the properties of agricultural low-carbon technologies. This is mainly attributed to the fact that when farmers are rich in household capital but have labor shortages, they tend to accept capital intensive, labor-saving technologies. When they are rich in land-labor resources, they tend to accept labor-increasing technologies. Risk averse farmers are more inclined to select low-risk technologies. The actual selection pathway is as follows: farmers’ perception of family properties → optimization of family property characteristics → selection of symmetric low-carbon technologies with consistent properties.

Zhang et al. [35] analyzed the selection behavior of agricultural low-carbon technologies from the perspective of the risk preference of farmers. The individual characteristics of farmers and their familiarity with externalities were significantly correlated with their risk preference. These were important factors affecting the selection of low-carbon agricultural technologies. However, this conclusion does not explain how risk preference affects the selection of low-carbon technologies with different properties. In contrast, this study classified low-carbon technologies according to their risk properties, and further found that there existed a significant constraining effect between family property characteristics and different low-carbon technologies. In other words, farmers with insufficient capital or properties are not able to adopt some technologies. If property constraints are not addressed, it is difficult to simply increase the intensity of outreach to improve the use of a certain technology. In particular, in the case of the increasingly significant differentiation of farmer livelihoods [36], it is difficult to break the initial barriers to enter into the market, which can increase the difficulty and effect of agricultural technology promotion.

## 6. Conclusions

This study focused on the selection, and factors impacting the selection, of low-carbon agricultural technologies by different types of farmers in the context of achieving carbon neutrality. The study generated a theoretical farmer technology selection model, involving technical constraints and risk properties. The model was then empirically tested by using a logistic model, employing survey data collected from farmers in Jiangsu, China. This illustrated the differences and factors impacting the selection of different low-carbon agricultural technologies by different farmers, further analyzing the influence of family properties on different low-carbon agricultural technologies. The main conclusions drawn from the results of this study are as follows:

(1) Large-scale farmers are more inclined to accept capital-intensive low-carbon technologies, such as new varieties, straw recycling, soil testing, and formulated fertilization. Mid-level part-time farmers are more inclined to accept capital-intensive, labor-saving, or low-risk low-carbon agricultural technologies. Low-level part-time farmers are inclined to accept labor-increasing technologies to reduce capital constraints and agricultural risks.
(2) The selection of low-carbon agricultural technologies of large-scale farmers and low-level part-time farmers is influenced by householder and plot characteristics, while mid-level part-time farmers are more influenced by plot characteristics. Specifically, from the perspective of the four low-carbon technologies (except the application of farmyard manure), the individual characteristics of large-scale farmers, especially age and education level, significantly impacted the adoption of low-carbon technologies. Plot characteristics generally negatively impacted the adoption of all four low-carbon technologies, in particular, the number of plots and land rent. Among the individual characteristics of mid-level part-time farmers, their health and education level exhibited lower negative impacts on some low-carbon technologies; however, other information regarding the head of the household played a positive role in promoting the application of the four low-carbon technologies, including whether the farmer was a land transfer farmer, as well as farmer health. For the low-level part-time farmers, only the number of plots was associated with increased adoption of farmyard manure application. Straw recycling by low-level part-time farmers was significantly positively impacted by whether or not the farmer was a land transfer farmer and the lease term. Whether the farmer was a land transfer farmer showed a greater impact, indicating that low-level part-time farmers with land transfer properties have a stronger preference for straw recycling technology.
(3) Households with capital constraints created by livelihood differentiations offer strong constraints with respect to adopting low-carbon agricultural technologies with capital intensive properties, such as straw recycling, new varieties, soil testing, and formulated fertilization. However, farmers with high land-labor resource constraints are more inclined to select labor-saving technologies, such as soil testing and formulated fertilization. Moreover, farmers with a stronger risk tolerance tend to accept high-risk technologies, such as new technologies and straw recycling technology. In other words, the selection of low-carbon agricultural technologies with different properties is significantly affected by the characteristics of family properties.

## Figures and Tables

**Table 1:** Properties and resource characteristics of different types of farmers.

Farmer Livelihood	Risk Preference	Labor Constraints	Capital Constraints
Large-scale farmers	Moderate: stronger risk resistance, higher risk due to the large land scale	Moderate: seasonal labor shortages, rising labor costs, stronger labor constraints	Moderate: have financial strength, but more capital is needed due to the large land scale
Mid-level part-time farmers	Low: stronger risk resistance	Strong: stronger labor constraints	Weak: low capital constraint due to higher degree of concurrent business
Low-level part-time farmers	High: lower risk resistance	Weak: weaker labor constraints	Strong: severe scarcity

**Table 2:** Possible choices of farmer livelihoods and agricultural low-carbon technologies.

	Benefits	Uncertainty	Labor Input	Capital Investment	Farmer Type That Selects This Technology
New varieties compared with older varieties	Low-carbon; increased or stabilized yields	Increase	Unchanged	Increased	Large-scale farmers; mid-level part-time farmers
Straw recycling compared with straw burning	Low-carbon; increased long term yields	Increase	Reduced	Increased	Large-scale farmers; mid-level part-time farmers
Soil testing and formulated fertilization compared with chemical fertilizer application	Low-carbon; increased long term yields	Reduce	Unchanged	Increased	Large-scale farmers; mid-level part-time farmers
Farmyard manure compared with chemical fertilizer	Low-carbon; increased yield and efficiency	Reduce	Increased	Reduced	Low-level part-time farmers

**Table 3:** Basic information of sample farmers.

	Variable	Mean	Standard Deviation
Household information	Age	52.65	9.26
	Education level	7.86	3.19
	Health level	1.15	0.419
	Village cadre or not	0.04	0.49
	Risk preference	0.33	0.34
	Land transfer farmer or not	0.67	0.47
Family characteristics	Labor resources	0.41	0.20
	Land resources	11.18	25.7
	Capital	21.34	20.74
	Land-labor resources	0.16	0.24
	Medium or large machinery or not	0.20	0.4

**Table 4:** Definition and descriptive statistics of independent variables.

	Variable	Definition and Description of Variables
Household information	Age	Age of head of household
	Education level	Number of years of formal education of head of household (years)
	Health level	Health status of the head of household
	Village cadre or not	Whether the head of household has been a village cadre or not; if yes = 1, if no = 0
	Risk preference	The value ranges from 0 to 1. A larger value indicates a higher risk
Family characteristics	Land transfer farmer or not	Has the farmer ever transferred land in; if yes = 1, if no = 0
	Labor resources	Proportion of agricultural labor compared to total household labor (%)
	Land resources	Cultivated land size (mu)
	Capital	Value of principal residence in 2021 (10 thousand yuan)
	Land-labor resources	The number of agricultural labor per mu
	Medium or large machinery or not	Whether there is large or medium machinery in the household
Plot characteristics	The lease term	Term of lease/year
	Land rent	Land rent per mu/year (yuan)
	The number of plots	The number of plots used
	Confirmed or not	Whether the land is confirmed; if yes = 1, if no = 0
Regional characteristics	Degree of circulation of village land	Proportion of cultivated land area participating in transfer compared to the total cultivated land in village in 2020 (%)
	Distance from village to town	Distance from the location of the village committee to the location of the village government (km)
	Intensity of technology popularization in the village	Number of technology promotion activities or observation tours held by agricultural technology stations or dealers in the village in 2020 (times)
	Agricultural machinery service stations or not in the village	Whether there are large and medium scale agricultural machinery service stations or not in the village; if yes = 1, if no = 0

**Table 5:** Farmer livelihood and selection of agricultural low-carbon technologies (%).

Farmer Livelihood	New Varieties (Capital Intensive–Labor Stabilizing–High-Risk)	Straw Recycling (Capital Intensive–Labor Saving–High-Risk)	Soil Testing and Formulated Fertilization (Capital Intensive–Labor Saving–Low-Risk)	Farmyard Manure Application (Capital Stabilizing–Labor Increasing–Low-Risk)
Large-scale farmers	57.14 *	74.28	24.28	11.54 *
Mid-level part-time farmers	52.43	71.35 *	8.1 **	15.13 **
Low-level part-time farmers	42.86 *	74.29	24.29	21.43 *

Note: this table counts the proportion of farmers adopting this technology in each group (different types of farmers’ livelihoods). * and ** indicate significant differences at the levels of 0.1 and 0.05, respectively (ANOVA).

**Table 6:** Farmer livelihood and selection of agricultural low-carbon technologies (based on low-level part-time farmers).

Variable	New Varieties (Capital Intensive–Labor Stabilizing–High-Risk)		Straw Recycling (Capital Intensive–Labor Saving–High-Risk)		Soil Testing and Formulated Fertilization (Capital Intensive–Labor Saving–Low-Risk)		Farmyard Manure Application (Capital Stabilizing–Labor Increasing–Low-Risk)	
Farmer livelihood	Coef	(dy/dx)	Coef	(dy/dx)	Coef	(dy/dx)	Coef	(dy/dx)
Large-scale farmers	0.84 * (1.66)	0.17 * (1.66)	3.07 *** (5.71)	0.61 *** (5.62)	0.75 * (1.69)	0.148 * (1.71)	−2.11 *** (−4.68)	−0.42 *** (4.77)
Mid-level part-time farmers	−0.02 (−0.05)	−0.004 (−0.04)	3.006 *** (5.86)	0.58 *** (4.88)	0.04 (0.13)	0.008 (0.15)	−3.39 *** (−7.28)	−0.66 *** (−6.83)
Village characteristics								
Distance from the village to town	−0.03 (−0.87)	−0.006 (−0.11)	0.65 (1.64)	0.13 (1.37)	−0.03 (−0.75)	0.006 (−0.90)	0.669 (1.19)	0.13 (1.38)
Intensity of agricultural machinery popularization in the village	1.46 * (1.81)	0.28 * (1.90)	1.41 * (1.76)	0.28 * (1.76)	1.26 ** (2.20)	0.25 ** (2.19)	−2.22 *** (−3.78)	−0.43 *** (−3.77)
Degree of circulation of village land	0.74 (0.51)	0.144 (0.50)	0.31 ** (2.11)	0.062 ** (2.21)	−0.06 (−0.76)	−0.012 (−0.77)	0.27 *** (3.87)	0.053 *** (4.12)
Agricultural machinery service stations or not	0.09 (0.79)	0.017 (0.80)	0.25 *** (4.16)	0.05 *** (4.10)	0.07 (0.98)	0.014 (0.10)	−0.001 (−0.24)	−0.0002 (−0.39)
Region (county)	Controlled							
Pseudo R^2^	0.15	—	0.16	—	0.02	—	0.27	—
Number of samples	307							

Note: *, **, and *** denote significant differences at 0.1, 0.05, and 0.01 levels, respectively. The regression results are Z values in parentheses.

**Table 7:** Large-scale farmers and selection of agricultural low-carbon technologies.

Variable	New Varieties (Capital Intensive–Labor Stabilizing–High-Risk)		Straw Recycling (Capital Intensive–Labor Saving–High-Risk)		Soil Testing and Formulated Fertilization (Capital Intensive–Labor Saving–Low-Risk)		Farmyard Manure Application (Capital Stabilizing–Labor Increasing–Low- Risk)	
Householder information	Coef	(dy/dx)	Coef	(dy/dx)	Coef	(dy/dx)	Coef	(dy/dx)
Age	0.35 * (1.81)	0.09 * (1.83)	0.25 (1.16)	0.06 (1.15)	0.04 (0.24)	0.250 (0.24)	0.61 (0.22)	0.15 (0.23)
Education level	0.14 *** (2.87)	0.25 *** (3.01)	0.02 ** (1.99)	0.005 ** (2.01)	1.13 (1.09)	0.27 (1.10)	−0.49 (−1.2)	−0.12 (−1.11)
Health level	0.11 (1.17)	0.03 (1.10)	0.01 (0.04)	0.002 (0.04)	0.57 *** (2.98)	0.11 *** (3.0)	−0.179 * (−1.77)	−0.04 * (−1.77)
Village cadre or not	0.22 *** (4.18)	0.05 *** (3.93)	0.13 (0.29)	0.25 (0.30)	0.05 * (1.78)	0.012 * (1.77)	0.02 (0.83)	0.005 (0.78)
Whether the farmer is a land transfer	0.09 (1.43)	0.02 (1.40)	0.13 (0.29)	0.03 (0.30)	0.04 ** (2.16)	0.01 ** (2.19)	−0.41 *** (−3.83)	−0.10 *** (−3.98)
Plot characteristics								
The lease term	0.08 *** (4.13)	0.02 *** (4.22)	0.005 (0.26)	0.001 (0.33)	0.24 *** (2.97)	0.06 *** (3.28)	−0.10 *** (−2.81)	−0.02 *** (−2.80)
Land rent	−0.10 (−0.82)	−0.03 (−0.81)	0.23 (0.80)	0.06 (0.98)	−0.001 (−0.74)	−0.0003 (−0.75)	−0.002 ** (−2.06)	−0.0005 ** (−2.08)
The number of plots	−0.02 (−0.25)	−0.005 (−0.24)	−0.31 *** (−3.93)	−0.08 *** (−4.0)	−0.33 * (−1.68)	−0.08 * (−1.69)	−0.01 (−0.86)	−0.08 (−0.90)
Region (county)	Controlled							
Pseudo R^2^	0.1577	—	0.3376	—	0.3729	—	0.1846	—
Number of samples	52							

Note: *, **, and *** denote significant differences at 0.1, 0.05, and 0.01 levels, respectively. The regression results are Z values in parentheses.

**Table 8:** Mid-level part-time farmers and selection of agricultural low-carbon technologies.

Variable	New Varieties (Capital Intensive–Labor Stabilizing–High-Risk)		Straw Recycling (Capital Intensive–Labor Saving–High-Risk)		Soil Testing and Formulated Fertilization (Capital Intensive–Labor Saving–Low-Risk)		Farmyard Manure Application (Capital Stabilizing–Labor Increasing–Low-Risk)	
Householder Information	Coef	(dy/dx)	Coef	(dy/dx)	Coef	(dy/dx)	Coef	(dy/dx)
Age	0.0004 (0.02)	0.0001 (0.03)	0.03 (1.13)	0.01 (1.09)	0.03 (0.64)	0.01 (0.64)	0.001 (1.27)	0.0003 (1.30)
Education level	0.065 ** (2.21)	0.002 ** (2.35)	0.005 (0.1)	0.002 (0.18)	0.07 (0.21)	0.02 (0.22)	−0.17 (−0.61)	−0.05 (−0.87)
Health level	0.414 (1.05)	0.13 (1.00)	−0.03 (−1.13)	−0.01 (−1.15)	0.16 (0.52)	0.05 (0.60)	0.17 (1.51)	0.05 (1.51)
Village cadre or not	0.04 (0.09)	0.01 (0.11)	0.40 (0.94)	0.01 (1.00)	0.11 ** (2.07)	0.03 ** (2.20)	0.01 (0.31)	0.003 (0.30)
Land transfer farmer or not	0.615 (1.41)	0.20 (1.48)	0.006 (0.07)	0.002 (0.12)	0.13 (0.16)	0.04 (0.20)	0.53 (0.66)	0.17 (0.66)
Plot characteristics								
The lease term	0.0008 (1.02)	0.0002 (1.02)	0.02 *** (3.51)	0.006 *** (3.60)	0.01 *** (3.36)	0.003 *** (3.40)	0.03 (1.43)	0.009 (1.44)
Land rent	−0.031 *** (−2.73)	−0.01 *** (−3.01)	−0.01 * (−1.79)	−0.12 * (−1.88)	−0.12 *** (−3.35)	−0.04 *** (−3.40)	0.0004 (0.81)	0.0001 (0.09)
The number of plots	−0.244 *** (−3.64)	−0.08 *** (−3.98)	−0.02 ** (−1.98)	−0.006 ** (−2.02)	−0.001 (−0.32)	−0.0003 (−0.34)	0.017 (0.51)	0.005 (0.61)
Constant	0.612 (0.48)	0.19 (0.48)	2.34 (1.50)	0.72 (1.55)	−0.09 (−0.39)	−0.03 (−0.40)	0.12 (0.35)	0.04 (0.39)
Region (county)	Controlled							
Pseudo R^2^	0.3810	—	0.2670	—	0.2918	—	0.1665	—
Numbers	185							

Note: *, **, and *** denote significant differences at 0.1, 0.05, and 0.01 levels, respectively. The regression results are Z values in parentheses.

**Table 9:** Low-level part-time farmers and selection of agricultural low-carbon technologies.

Variable	New Varieties (Capital Intensive–Labor Stabilizing–High-Risk)		Straw Recycling (Capital Intensive–Labor Saving–High-Risk)		Soil Testing and Formulated Fertilization (Capital Intensive–Labor Saving–Low-Risk)		Farmyard Manure Application (Capital Stabilizing–Labor Increasing–Low-Risk)	
Householder information	Coef	(dy/dx)	Coef	(dy/dx)	Coef	(dy/dx)	Coef	(dy/dx)
Age	−0.01 −(0.42)	−0.002 (−0.4)	0.04 (0.65)	0.008 (0.80)	0.003 (0.41)	0.0005 (0.45)	0.03 (0.80)	0.006 (0.81)
Education level	0.447 (0.71)	0.086 (−0.80)	0.04 (0.27)	0.008 (0.27)	0.03 (1.55)	0.006 (1.60)	0.15 (1.44)	0.03 (1.24)
Health level	0.07 (0.11)	0.01 (0.54)	0.75 (0.87)	0.14 (0.87)	0.31 (1.15)	0.06 (1.31)	−0.69 (−0.48)	−0.13 (−0.64)
Village cadre or not	0.66 (1.57)	0.13 (1.45)	0.07 (0.08)	0.013 (0.08)	0.12 (0.82)	0.02 (0.90)	0.46 (0.66)	0.09 (0.70)
Land transfer farmer or not	0.259 (0.65)	0.05 (0.72)	0.28 * (1.73)	0.05 * (1.88)	0.13 (0.97)	0.03 (1.02)	−0.07 (−0.88)	−0.01 (−0.90)
Plot characteristics								
The lease term	0.23 (0.65)	0.05 (0.66)	0.039 *** (3.08)	0.01 *** (3.40)	0.01 (0.56)	0.002 (0.76)	−1.46 (−1.50)	−0.28 (−1.50)
Land rent	0.14 (0.33)	0.03 (0.43)	−0.039 (−1.08)	−0.01 (−1.10)	−0.227 *** (−3.91)	−0.04 *** (−4.01)	−0.37 (−0.56)	−0.07 (−0.56)
The number of plots	−0.101 (−0.24)	−0.02 (−0.34)	−0.11 (−0.64)	−0.02 (−0.79)	−0.001 (−1.14)	−0.0002 (−1.24)	0.09 *** (3.53)	0.02 *** (4.33)
Region (county)	Controlled							
Pseudo R^2^	0.1325	—	0.1625	—	0.2255	—	0.1153	—
Numbers	70							

Note: *, **, and *** denote significant differences at 0.1, 0.05, and 0.01 levels, respectively. The regression results are Z values in parentheses.

**Table 10:** Characteristics of farmer properties and selection of agricultural low-carbon technologies with different properties.

	Straw Recycling(Capital Intensive–Labor Saving–High-Risk)		New Varieties(Capital Intensive–Labor Stabilizing–High-Risk)		Farmyard Manure Application(Capital Stabilizing–Labor Increasing–Low-Risk)		Soil Testing and Formulated Fertilization(Capital Intensive–Labor Saving–Low-Risk)	
Characteristics of farmer properties	Coef	(dy/dx)	Coef	(dy/dx)	Coef	(dy/dx)	Coef	(dy/dx)
Capital	0.063 *** (3.37)	0.01 *** (3.36)	0.112 ** (−4.39)	0.02 ** (−4.27)	−0.859 ** (2.06)	−0.147 ** (2.07)	0.669 * (1.75)	0.13 * (1.80)
Risk preference	−0.035 (−1.29)	−0.007 (−1.30)	−0.08 *** (−3.64)	−0.02 *** (−3.62)	−0.157 (−0.12)	−0.03 (−0.12)	0.017 (1.62)	0.003 (1.60)
Land-labor resources	−0.198 *** (−4.22)	−0.04 *** (−4.22)	−0.028 (−0.77)	−0.005 (−0.80)	2.82 ** (2.01)	0.56 ** (1.99)	−0.001 ** (2.48)	−0.002 ** (2.52)
Control variable	Controlled							
Region (county)	Controlled							
Pseudo R^2^	0.73	—	0.46	—	0.21	—	0.28	—
Number of samples	280							

Note: *, **, and *** denote significant differences at 0.1, 0.05, and 0.01 levels, respectively. The regression results are Z values in parentheses.

## Data Availability

Materials and anonymous data are available from the authors on request.
